# Age-adjusted eosinophilia in preterm infants with feeding intolerance: a retrospective cohort study

**DOI:** 10.1186/s12876-026-05093-w

**Published:** 2026-07-22

**Authors:** Ozge Serce Pehlevan, Merve Cekildas, Ayla Gunlemez

**Affiliations:** 1 Department of Neonatology, Division of Pediatrics, Kocaeli University Faculty of Medicine, Kocaeli, Turkey; 2 Kocaeli University School of Medicine, Kocaeli, Turkey

**Keywords:** Allergic enterocolitis, cow’s milk protein allergy, eosinophilia, feeding intolerance, necrotizing enterocolitis

## Abstract

**Purpose:**

Feeding intolerance (FI) remains diagnostically challenging because of nonspecific symptoms and overlap with multiple gastrointestinal conditions. This study investigated the association between peripheral eosinophilia and FI in preterm infants with exploratory evaluation of necrotizing enterocolitis (NEC) and cow’s milk protein–related gastrointestinal disease in relation to eosinophilia.

**Methods:**

In this retrospective cohort study, 122 preterm infants (< 34 weeks’ gestation, < 2000 g) with eosinophil counts ≥ 700/mm³ were included. Infants were subsequently reclassified according to gestational- and postnatal-age–adjusted eosinophil reference values. Clinical features of FI, NEC, and cow’s milk protein–related gastrointestinal disease were compared between infants with and without age-adjusted eosinophilia. Multivariable logistic regression was used to explore clinical variables independently associated with feeding intolerance.

**Results:**

Age-adjusted eosinophilia was present in 100 infants. FI was more frequent in eosinophilic infants (86% vs. 63.6%, *p* = 0.027), with longer feeding interruption (median 2 vs. 0.5 days, *p* = 0.028), more abdominal distension (60% vs. 31.8%, *p* = 0.019), and prolonged hospitalization (60 vs. 41 days, *p* = 0.034). In the primary multivariable model, age-adjusted eosinophilia was independently associated with feeding intolerance (OR 6.00; 95% CI 1.32–27.26; *p* = 0.020), although the association was attenuated in sensitivity analysis (OR 2.87; 95% CI 0.86–9.58; *p* = 0.087). All 13 NEC cases occurred in eosinophilic infants, although the temporal relationship between eosinophilia and NEC varied. Suspected cow’s milk protein–related gastrointestinal disease was identified in 11 infants, all of whom showed clinical improvement following dietary elimination.

**Conclusions:**

Age-adjusted eosinophilia was frequently observed in preterm infants with FI and more severe gastrointestinal manifestations. While unlikely to be causally related to FI, it may reflect an accompanying immune or inflammatory response during gastrointestinal disease. Eosinophil counts may provide supportive information in selected infants with persistent FI or NEC-like presentations, particularly when alternative inflammatory gastrointestinal processes are being considered.

**Supplementary Information:**

The online version contains supplementary material available at https://doi.org/10.1186/s12876-026-05093-w.

## What is known

- Feeding intolerance and necrotizing enterocolitis share overlapping and nonspecific clinical features in preterm infants.
- Peripheral eosinophilia has been intermittently reported in non–IgE-mediated gastrointestinal disorders including cow’s milk protein–related enterocolitis and food protein–induced enterocolitis syndrome in this population.

## What is new

- Reclassification using gestational- and postnatal-age–adjusted eosinophil reference ranges was associated with a subgroup of preterm infants demonstrating feeding intolerance and more severe gastrointestinal manifestations.
- Eosinophilia appears to accompany rather than initiate gastrointestinal disease and may represent a contextual immune signal observed in selected infants with persistent feeding intolerance or NEC-like presentations.

## Introduction

The reported prevalence of feeding intolerance in preterm infants varies widely with recent studies reporting rates between 30% and 60% depending on gestational age and feeding protocols [1, 2]. Feeding intolerance encompasses a broad spectrum of clinical manifestations including feeding interruption, abdominal distension, increased gastric residuals, vomiting, and changes in stool patterns. These findings are inherently nonspecific and may reflect conditions ranging from transient gastrointestinal immaturity to serious diseases such as necrotizing enterocolitis (NEC). This considerable clinical overlap poses a persistent diagnostic challenge in the neonatal intensive care setting.

NEC is one of the most severe gastrointestinal emergencies in the neonatal period predominantly affecting preterm infants. Its pathogenesis involves a complex interplay of intestinal immaturity, dysbiosis, hypoxia–ischemia, and early enteral feeding leading to exaggerated inflammatory injury [3]. Early clinical signs of NEC are often indistinguishable from those of benign feeding intolerance frequently resulting in diagnostic uncertainty and variability in management strategies [3].

In recent years, increasing attention has been directed toward non-NEC causes of feeding intolerance particularly cow’s milk protein–related non–IgE-mediated gastrointestinal disorders. These conditions may resemble NEC in clinical presentation and radiologic findings, potentially leading to diagnostic uncertainty and prolonged or unnecessary interventions [4–7]. Although classical food protein–induced enterocolitis syndrome (FPIES) is considered uncommon in premature neonates, isolated reports have described NEC-like presentations associated with cow’s milk protein exposure in this population [4–7]. Notably, trace amounts of cow’s milk proteins ingested by the mother may be detectable in breast milk and have been proposed as a potential trigger for allergic gastrointestinal reactions in susceptible preterm infants although the transferred amounts are believed to be very low [8, 9].

Eosinophils play key roles in allergic inflammation and host defense and are highly concentrated within the gastrointestinal mucosa. Upon activation, they release cytotoxic granule proteins and inflammatory mediators capable of inducing mucosal injury [10]. Several case reports and small series have described peripheral eosinophilia in preterm infants presenting with gastrointestinal symptoms suggestive of cow’s milk protein–related gastrointestinal disease [8, 11]. However, the clinical interpretation of eosinophilia in neonates remains challenging, as eosinophil counts vary substantially according to gestational and postnatal age, limiting the reliability of fixed diagnostic thresholds [11–14].

Despite growing recognition of the overlap between NEC and food protein–related enterocolitis, the relevance of applying gestational- and postnatal-age–adjusted eosinophil reference ranges in preterm infants with feeding intolerance has not been systematically evaluated. The present study aimed to examine feeding intolerance in preterm infants born at < 34 weeks’ gestation by applying age-adjusted eosinophil reference values after initial identification using a fixed eosinophilia threshold, and to explore the clinical characteristics associated with eosinophilia in this population.

## Materials and methods

This retrospective cohort study was conducted in the neonatal intensive care unit (NICU) of a tertiary care hospital and was approved by the Institutional Ethics Committee. Preterm infants with a gestational age < 34 weeks and a birth weight < 2000 g who were admitted between January 2019 and January 2025 were eligible for inclusion. Infants with major congenital anomalies (e.g., gastrointestinal atresia, abdominal wall defects or Hirschsprung disease), congenital metabolic disorders, death within the first postnatal week or incomplete clinical or laboratory data were excluded.

Infants were followed throughout their NICU hospitalization until discharge, transfer, or death. Eosinophil measurements were obtained as part of routine serial clinical monitoring, typically through serial complete blood counts performed approximately every 7–10 days during hospitalization with additional testing when clinically indicated. Initially, a fixed threshold of ≥ 0.7 × 10⁹/L was used to identify infants with elevated eosinophil counts and to define the study cohort, consistent with prior neonatal eosinophilia reports [11, 12].

Infants identified using this fixed eosinophil cutoff were subsequently reclassified according to gestational- and postnatal-age–specific eosinophil reference ranges derived from Christensen et al. and Henry et al. [13, 14] (Supplementary Table S1). For each infant, eosinophil counts were interpreted based on the corresponding gestational age and postnatal week at the time of blood sampling. Infants with eosinophil values exceeding the upper reference limit for their gestational and postnatal age category were classified as having age-adjusted eosinophilia, whereas those remaining within the reference intervals were categorized as non-eosinophilic. Each category was further subdivided according to the presence or absence of gastrointestinal intolerance, resulting in four analytical subgroups.

Demographic and perinatal variables included gestational age, birth weight, sex, mode of delivery, and 5-minute Apgar score. Maternal characteristics such as chorioamnionitis, gestational diabetes, hypertensive disorders of pregnancy, and antenatal steroid exposure were recorded. Feeding-related data encompassed feeding type, use of human milk fortifier or protein supplementation, initiation and advancement of enteral feeding, and duration of feeding interruption. Donor human milk was not available at our institution during the study period. Enteral feeding therefore consisted primarily of maternal breast milk, preterm formula, and, when clinically indicated, extensively hydrolyzed or amino acid–based formulas.

Gastrointestinal intolerance was defined as the presence of clinical, radiologic, feeding-related, or systemic findings suggestive of impaired enteral tolerance. These included abnormal gastric aspirates (blood-stained, discolored, or increased residuals), abdominal distension or tenderness, abnormal bowel sounds, ileus, feeding interruption or withholding. Systemic manifestations such as apnea, bradycardia, or shock temporally associated with feeding were also considered supportive findings. NEC was diagnosed in accordance with national guidelines and established consensus criteria, based on integrated assessment of clinical findings, laboratory abnormalities, and characteristic radiologic features, and was staged using the Modified Bell Staging System [3, 15]. Sepsis was defined by the presence of two or more clinical signs (including apnea, lethargy, temperature instability, feeding intolerance, or hypotension) together with abnormal inflammatory markers (elevated C-reactive protein or procalcitonin, abnormal white blood cell count, or thrombocytopenia), prompting initiation of antibiotic therapy regardless of culture results [16]. To minimize misclassification with spontaneous intestinal perforation, NEC cases were adjudicated based on combined clinical course and radiographic criteria, and surgical findings when available; cases presenting with isolated pneumoperitoneum without pneumatosis intestinalis/portal venous gas were reviewed for spontaneous intestinal perforation.

The diagnosis of cow’s milk protein allergy (CMPA) was based on gastrointestinal symptoms (vomiting, diarrhea, abdominal distension, hematochezia), dermatologic or respiratory findings when present, and improvement following elimination of cow’s milk protein in accordance with ESPGHAN recommendations [17]. Fecal occult blood testing was performed selectively in clinically suspicious cases, as routine interpretation may be confounded by orogastric tube–related mucosal irritation in preterm infants. Rechallenge test was not performed.

The primary outcome was the association between gestational- and postnatal-age–adjusted eosinophilia and gastrointestinal intolerance among preterm infants identified using an initial fixed eosinophil cutoff. Secondary outcomes included characterization of feeding intolerance features, evaluation of NEC and suspected cow’s milk protein–related gastrointestinal disease frequency, and assessment of their relationship with eosinophilia.

### Statistical analysis

Statistical analyses were conducted using IBM SPSS Statistics, version 25 (IBM Corp., Armonk, NY, USA). Normally distributed data are presented as mean ± standard deviation (SD), non-normally distributed data as median (IQR), and categorical variables as counts and percentages. Group comparisons were made using the Student’s t-test or Mann–Whitney U test for continuous variables, and the chi-square or Fisher’s exact test for categorical variables. To explore clinical variables independently associated with feeding intolerance, multivariable logistic regression analysis was performed. Variables with clinical relevance or *p* < 0.10 in univariable analyses were included in the model. A parsimonious sensitivity model, limited to key covariates (gestational age, sepsis, and age-adjusted eosinophilia), was constructed to test the robustness of associations. Results are reported as adjusted odds ratios (OR) with 95% confidence intervals (CI). A two-sided *p* < 0.05 was considered statistically significant.

## Results

### Study population and eosinophilia incidence

A total of 757 infants (gestational age < 34 weeks and birth weight < 2000 g) were admitted to the NICU between January 2019 and 2025. Eosinophil counts *≥* 700 cells/mm³ were observed in 166 (21.9%) among these infants. Infants with major congenital anomalies (*n* = 14), congenital metabolic disorders (*n* = 8), incomplete clinical or laboratory data (*n* = 7), or early neonatal death within the first postnatal week (*n* = 15) were excluded. After exclusions, 122 infants comprised the study cohort; among these, age-adjusted eosinophilia was confirmed in 100 (82%). All infants received maternal breast milk during hospitalization. Exclusive maternal breast milk feeding was observed in 55 infants (45.1%), whereas 43 infants (35.2%) received maternal breast milk for 50–99% of enteral intake and 24 infants (19.7%) received maternal breast milk for less than 50% of enteral intake.

### Eosinophilia and perinatal characteristics

The median day of occurrence of eosinophilia was 23 days (IQR: 7.4) ranging from day 4 to day 49. The median number of eosinophilic episodes per infant was 2 (IQR: 2) with a maximum of 6. The median peak eosinophil count was 1.75 × 10⁹/L (IQR: 1.0–4.7 × 10⁹/L). The cumulative distribution of eosinophilia by week of life showed a progressive increase: 22% of cases occurred in the first week, 24% in the second, 46% in the third, and 58% in the fourth; eosinophilia persisted beyond the fifth week in 36% of infants.

When infants were grouped based on the presence or absence of eosinophilia, no significant differences between groups were observed for gestational age or birth weight. Among maternal factors, preeclampsia was more frequent in the non-eosinophilic group (54.3% vs. 32%, *p* = 0.046). There were no significant differences in other perinatal characteristics between the groups (Table 1).

**Table 1:** Characteristics of infants with and without eosinophilia

	Eosinophilia (+) *N* = 100	Eosinophilia (–) *N* = 22	*p*-value
Demographics			
Gestational age (weeks), median (IQR)	29(4)	30(4)	0,62
Birth weight (g), median (IQR)	1180(695)	1255(495)	0,23
Small for gestational age, n (%)	13(13)	3 (13,6)	1
Maternal factors, n(%)			
Preeclampsia	32(32)	12(54,3)	0,046
PROM > 18 h ± chorioamnionitis*	19(19)	2(9,1)	0,36
Antenatal steroid	65(65)	18(82)	0,20
Oligohydramnios	18(18)	6(27,3)	0,37
Neonatal Morbidities			
Patent ductus arteriosus, n(%)	47(47)	6(27,3)	0,09
Sepsis, n(%)	68(68)	11(50)	0,10
Hospitalization (days), median(IQR)	60(50)	41(27)	0,034
Feeding Characteristics			
Minimal enteral feeding initiation (days), median (IQR)	1(2)	1(1)	0,30
Bovine fortifier initiation (days), median (IQR)	14(15)	14(14)	0,69
Protein supplementation initiation (days), median (IQR)	25(15)	30(13)	0,33
Human milk intake < 50%, n(%)	59(59)	9(40,9)	0,25
Elimination diet			
Infant (Amino acid–based formula)	8 (8)	0	0,20
Mother (Dairy-free diet)	9(9)	0	0,21

*PROM Premature rupture of membranes

### Feeding intolerance

In the overall cohort (*n* = 122), feeding intolerance occurred in 82% of infants during hospitalization, while 40% demonstrated feeding intolerance temporally associated with documented eosinophilia episodes. Feeding intolerance during hospitalization was significantly more frequent in the eosinophilic group (86% vs. 64%, *p* = 0.027). However, the median day of onset did not differ significantly [8 (IQR:17) vs. 9 (IQR:13), *p* = 0.97)]. The duration of feeding interruption was longer in eosinophilic infants [2 days (IQR: 8) vs. 0.5 days (IQR: 2), *p* = 0.028)]. Vomiting was more common in the eosinophilic group (47% vs. 27.3%), although this difference did not reach statistical significance (*p* = 0.10). In contrast, abdominal distension and/or tenderness was significantly more frequent among eosinophilic infants (60% vs. 31.8%; *p* = 0.019). In addition, the duration of hospitalization was significantly longer in infants with eosinophilia [60 days (IQR: 50) vs. 41 days (IQR: 27)], *p* = 0.034). Additional clinical parameters related to feeding intolerance are summarized in Table 2.

**Table 2:** Characteristics of feeding intolerance by eosinophilia status

	Eosinophilia (+) *N* = 100	Eosinophilia (–) *N* = 22	*p*-value
Feeding intolerance during hospitalization, *n*(%)	86(86)	14(14)	0,027
Feeding intolerance initiation (days), median (IQR)	8(17)	9(13)	0,97
Duration of feeding interruption (days), *median (IQR)*	2(8)	0,5 (2)	0,028
Vomiting, *n(%)*	47(47)	6(27,3)	0,10
Abdominal distension ± tenderness, *n(%)*	60(60)	7(31,8)	0,019
Residuals, *n(%)*	46(46)	8(36,4)	0,48
Rectal bleeding, *n(%)*	11(11)	1(4,5)	0,69
No spontaneous stool passage, *n(%)*	23(23)	5(22,7)	0,97
Abdominal wall erythema, crepitus, induration, *n(%)*	17(17)	1(4,5)	0,36
Apnea ± acute respiratory failure associated with feeding, *n(%)*	66(66)	12(54)	0,33
Hypotension, *n(%)*	17(17)	1(4,5)	0,19
Symptomatic gastrointestinal therapy*, *n(%)*	58(58)	10(45,5)	0,28

*Includes probiotics, clarithromycin, domperidone, and alginate-based reflux suppressant

In the multivariable analysis, two models were constructed to explore clinical variables associated with feeding intolerance. In the primary model (including all clinical covariates), age-adjusted eosinophilia was independently associated with feeding intolerance (OR 6.00, 95% CI 1.32–27.26; *p* = 0.020). However, due to the wide confidence intervals, a refined sensitivity model was developed to prioritize parsimony. In this second model, gestational age and late-onset sepsis remained independently associated with feeding intolerance, while age-adjusted eosinophilia showed an attenuated association in the same direction (OR 2.87, 95% CI 0.86–9.58; *p* = 0.087) (Table 3). As an additional sensitivity analysis, infants who developed eosinophilia during the postoperative period following NEC surgery (*n* = 3) were excluded. The results remained essentially unchanged, with age-adjusted eosinophilia showing a similar association with feeding intolerance (OR 2.85, *p* = 0.089), supporting the robustness of the findings.

**Table 3:** Multivariable logistic regression analysis for factors associated with feeding intolerance

Variable	Primary model Adjusted OR (95% CI)	*p* value	Sensitivity model Adjusted OR (95% CI)	*p* value
Age-adjusted eosinophilia	6.00 (1.32–27.26)	0.020	2.87 (0.86–9.58)	0.087
Gestational age (weeks)	0.73 (0.55–0.96)	0.026	0.72 (0.56–0.93)	0.012
Late-onset sepsis	3.63 (1.53–8.62)	0.003	3.96 (1.71–9.19)	0.001
Preeclampsia	9.09 (1.66–49.88)	0.011	—	—
Patent ductus arteriosus	2.24 (0.58–8.65)	0.242	—	—

### Necrotizing enterocolitis and cow’s milk protein allergy

**Fig. 1:**
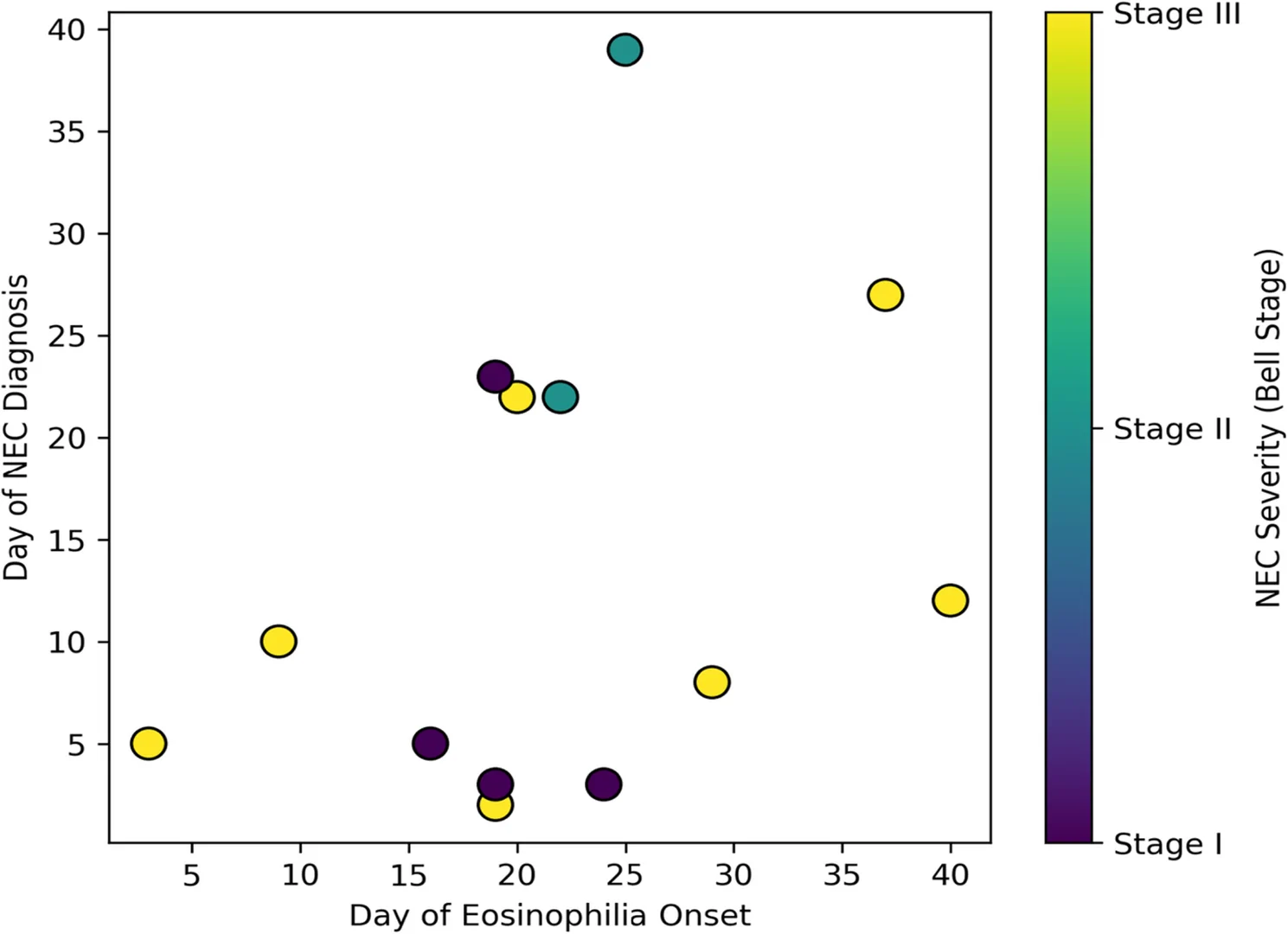
Timing of eosinophilia in relation to necrotizing enterocolitis diagnosis

All NEC cases (*n* = 13) occurred in the eosinophilic group. The median time to NEC diagnosis was 8 days after birth (IQR: 19 days). Of these, 7 cases were classified as Bell stage III, 2 cases as stage II, and 4 cases as stage I according to Modified Bell criteria. Eosinophilia was identified in different temporal associations with the onset of NEC. In 8 of 13 NEC cases, eosinophilia was observed concurrently with the initial presentation of NEC symptoms. In 3 cases, eosinophilia developed during the postoperative period together with ongoing gastrointestinal intolerance symptoms. In two infants initially diagnosed with NEC, persistent feeding intolerance accompanied by eosinophilia prompted consideration of cow’s milk protein–related enterocolitis, and both showed clinical improvement following dietary elimination. In these infants, eosinophilia had been present since symptom onset but was recognized only shortly thereafter. In addition, one infant demonstrated persistent eosinophilia together with NEC findings, although no clinical documentation suggesting suspicion of eosinophilia-associated gastrointestinal disease or FPIES was identified in the medical records (Fig. 1).

**Fig. 2:**
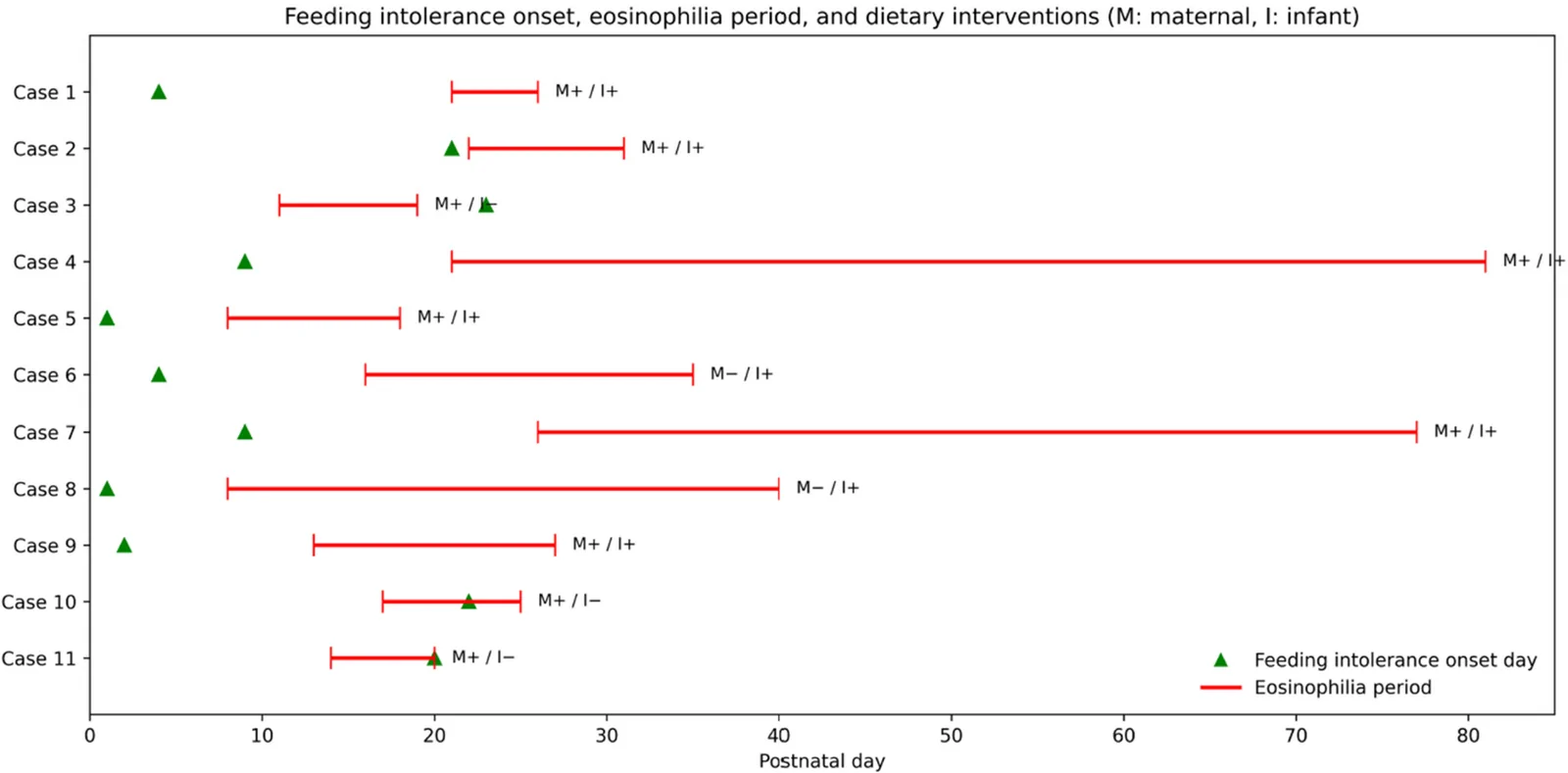
Eosinophilia episodes in relation to gastrointestinal intolerance according to cow’s milk protein allergy management strategy. Infants were followed throughout NICU hospitalization; the extended timeline reflects a small number of infants with prolonged hospitalization up to approximately postnatal day 80

Among the 100 infants with eosinophilia, 11 infants were suspected of having cow’s milk protein–related gastrointestinal disease. Maternal elimination diet was implemented for 9% of cases, while amino acid-based formula was initiated in 8% of infants with suspected food protein intolerance in the eosinophilic group. All showed clinical improvement following dietary modification (Fig. 2).

### Subgroup analysis

Following stratification into four subgroups based on eosinophilia and feeding intolerance, significant differences between subgroups were observed in gestational age and birth weight. Bonferroni-adjusted post hoc analysis revealed that birth weight differed significantly between eosinophilia-positive infants with and without feeding intolerance (1145 vs. 1795 g, *p* = 0.002). Gestational age also differed significantly between eosinophilic/feeding intolerant infants and non-eosinophilic feeding tolerant infants (29 vs. 32 weeks, *p* = 0.007). withoutBonferroni-corrected analysis also showed that the frequency of maternal preeclampsia was significantly higher in the non-eosinophilic feeding-intolerant group than in all other groups, and higher in the eosinophilic/feeding-intolerant group than in the eosinophilic/feeding-tolerant group (36% vs. 7.1%, *p* < 0.0083). Group-wise comparisons of demographic and clinical characteristics are presented in Table 4.

**Table 4:** Baseline characteristics of infants with/without eosinophilia and/or feeding intolerance

	Eosinophilia (+)		Eosinophilia (-)		
	Gastrointestinal intolerance (+) *N* = 86 (86%)	Gastrointestinal intolerance (–) *N* = 14 (14%)	Gastrointestinal intolerance (+) *N* = 14 (63,6%)	Gastrointestinal intolerance (–) *N* = 8(36,4%)	Over all *p*-value
Gestational age (weeks), median (IQR)	29 (3)	31 (1)	29 (3)	32 (5)	0,003
Birth weight (g), median (IQR)	1145 (500)	1795 (714)	1190 (493)	1430 (745)	0,011
Male sex, n (%)	39 (45,3)	7(50)	10 (71,4)	4 (50)	0,34
Small for gestational age, n (%)	12 (14)	1 (7,1)	1 (7,1)	2 (25)	0,60
APGAR 5th minute, median (IQR)	7(2)	7 (2)	8 (2)	8 (2)	0,054
Maternal factors, n (%)					
Preeclampsia	31 (36)	1 (7,1)	10 (71,4)	2 (25)	0,003
PROM > 18 h± chorioamnionitis	16 (18,6)	3 (21,4)	2 (14,3)	0	0,64
Antenatal steroid	56 (65,1)	9(64,3)	12(85,7)	6(75)	0,71
Oligohydroamnios	15(17,4)	3(21,4)	5(35,7)	1(12,5)	0,40

*PROM* premature rupture of membranes. P values represent overall comparisons across the four analytical subgroups using Kruskal–Wallis or chi-square/Fisher’s exact tests, as appropriate

## Discussion

Feeding intolerance remains a frequent yet diagnostically challenging problem in preterm infants. In this study, feeding intolerance was observed in 82% of infants classified as eosinophilic using gestational- and postnatal-age–adjusted reference values, and in 86% when a fixed eosinophil cutoff (≥ 700/mm³) was applied. These rates are substantially higher than the 30–60% prevalence reported in unselected preterm populations, where eosinophil status has not been considered [1]. This difference likely reflects both the high-risk nature of the study population (< 34 weeks’ gestation, < 2000 g). In addition, the wide variability in reported feeding intolerance rates across studies is partly attributable to the lack of a standardized definition of feeding intolerance in the neonatal literature. Notably, although feeding intolerance occurred in 82% of the cohort, only 40% demonstrated feeding intolerance temporally associated with documented eosinophilia. This finding supports the interpretation that eosinophilia accompanies only a subset of gastrointestinal disease episodes and is unlikely to represent a universal marker of feeding intolerance.

Although feeding intolerance is frequently attributed to gastrointestinal immaturity, it may also represent early manifestations of NEC or non–IgE-mediated allergic gastrointestinal disorders. Feeding intolerance occurred more frequently among infants with age-adjusted eosinophilia and was associated with more severe clinical features including abdominal distension, prolonged feeding interruption, and longer hospitalization in our cohort. Importantly, multivariable analyses demonstrated that the association between eosinophilia and feeding intolerance was attenuated in the reduced sensitivity model but remained directionally consistent. Although the direction and magnitude of the association remained largely unchanged in sensitivity analyses, the confidence intervals were wide and statistical significance was not consistently maintained, suggesting limited statistical power and the need for confirmation in larger prospective cohorts. Accordingly, these findings should be interpreted cautiously and considered hypothesis-generating rather than confirmatory.

The temporal relationship between eosinophilia and gastrointestinal symptoms was heterogeneous. In several cases, feeding intolerance preceded the detection of eosinophilia, and the observed dissociation between the median onset of feeding intolerance (day 8) and the subsequent peak of age-adjusted eosinophilia (day 23) further suggests that eosinophilia is more likely to emerge during the course of gastrointestinal disease than to represent an initiating event. A notable observation was that all NEC cases occurred exclusively within the eosinophilic group, despite no significant differences in traditional NEC risk factors such as gestational age, birth weight, patent ductus arteriosus, or late-onset sepsis. The relatively high NEC frequency likely reflects both the enriched high-risk cohort selected on the basis of eosinophilia and the tertiary referral nature of our center, which manages a large number of high-risk pregnancies and very preterm infants. Nevertheless, the exclusive occurrence of NEC within the eosinophilic group suggests that peripheral eosinophilia may identify infants with a lower threshold for severe intestinal inflammation.

Among infants in whom eosinophilia emerged after NEC surgery, eosinophilia developed a median of approximately 14 days following surgical intervention, supporting the possibility that postoperative inflammatory or immune responses contributed to late eosinophilia in a subset of cases. In two infants initially diagnosed with NEC, recognition of concurrent feeding intolerance and eosinophilia prompted dietary elimination, resulting in clinical improvement consistent with cow’s milk protein–related allergic enterocolitis. These observations suggest that eosinophilia may be underrecognized in routine NICU practice.

Furthermore, NEC onset in our cohort occurred earlier than classically reported. The median day of NEC diagnosis was 8 (range: 2–39), compared with the commonly reported onset window of 2–4 weeks of life in preterm infants [18]. The predominance of early-onset NEC may further support the possibility of pre-existing intestinal immune activation or sensitization in this population. Such activation may be influenced by early exposure to dietary antigens or ongoing inflammatory processes within the immature gut. On the other hand, given the early timing of several NEC diagnoses, misclassification with spontaneous intestinal perforation cannot be fully excluded in a retrospective design, particularly in cases staged as Bell III based on pneumoperitoneum. However, case adjudication incorporated radiographic features (pneumatosis/portal venous gas) and operative findings when available to reduce this risk.

Within the age-adjusted eosinophilic cohort, CMPA was suspected in 11 infants. Targeted nutritional interventions including maternal elimination diets or amino acid–based formulas resulted in clinical improvement in all cases, consistent with previous reports supporting dietary modification in selected infants with suspected allergic gastrointestinal disease [9]. Notably, infants who developed eosinophilia postoperatively frequently required extensively hydrolyzed or amino acid–based formulas to achieve feeding tolerance, underscoring the need for heightened awareness of immune-mediated feeding intolerance in this setting. Previous studies have linked peripheral eosinophilia in preterm infants to allergic gastrointestinal conditions, gastrointestinal morbidity, and adverse intestinal outcomes, supporting the interpretation that eosinophilia reflects a broader inflammatory response rather than a disease-specific process [8, 11, 12, 19–22]. Eosinophils are abundant within the gastrointestinal mucosa and release cytotoxic granule proteins and inflammatory mediators capable of inducing mucosal injury [10]. Supporting this association, Kim et al. reported significantly higher eosinophil percentages in infants with food protein–related enterocolitis compared with NEC, and elevated eosinophil levels have also been described in preterm NEC cases [4]. Our findings extend this literature by demonstrating that eosinophilia frequently accompanies feeding intolerance and NEC-like presentations in preterm infants, although its clinical relevance appears to be context dependent.

The higher frequency of preeclampsia in the non-eosinophilic group was an unexpected finding. However, the relationship between maternal hypertensive disorders and neonatal eosinophil responses remains poorly understood, and this observation should be interpreted cautiously as it may reflect residual confounding related to prematurity severity or other perinatal characteristics.

From a clinical perspective, eosinophilia should not be interpreted as a stand-alone diagnostic marker for NEC and should not by itself prompt interruption of enteral feeding or empiric formula modification. In preterm infants with acute systemic illness, abdominal tenderness, bloody stools, or radiologic abnormalities, standard NEC evaluation and management should remain the priority regardless of eosinophil count. However, eosinophilia may provide clinically meaningful contextual information when it is persistent, recurrent, or temporally associated with ongoing gastrointestinal symptoms. In infants with persistent or recurrent feeding intolerance, postoperative gastrointestinal symptoms, abdominal distension, or NEC-like presentations in whom sepsis and NEC are not clearly established, concurrent or persistent eosinophilia may prompt reassessment of feeding exposures and consideration of alternative inflammatory gastrointestinal processes, including possible cow’s milk protein–related enterocolitis. In such selected cases, careful review of feeding exposures, including bovine milk–based fortifiers or formulas, together with assessment of the clinical response to dietary modifications, may be informative. Interpretation should therefore incorporate gestational maturity, symptom chronology, feeding exposure history, and concurrent evaluation for NEC or sepsis.

A major strength of this study was the use of both a conventional fixed eosinophil cutoff for cohort identification and gestational- and postnatal-age–adjusted reference ranges for classification, improving comparability with previous studies while reducing potential misclassification. Additional strengths include detailed longitudinal clinical assessment within a high-risk preterm NICU cohort and evaluation of the temporal relationship between eosinophilia, feeding intolerance, and NEC-like gastrointestinal manifestations in routine clinical practice.

However, several limitations should be acknowledged. First, the retrospective single-center design may limit generalizability and introduces the possibility of residual confounding related to illness severity, mechanical ventilation, antibiotic exposure, transfusions, surgical interventions, and dynamic feeding practices. Second, feeding intolerance in premature infants also remains a clinically heterogeneous condition without universally standardized diagnostic criteria, and overlap with sepsis, NEC, intestinal immaturity, and other gastrointestinal disorders may therefore have contributed to diagnostic misclassification. Third, additional testing in clinically unstable infants may have introduced sampling variability and bias toward sicker patients. Furthermore, because infants were initially identified using a fixed eosinophil cutoff and subsequently reclassified using higher age-adjusted reference thresholds, the final study population was enriched for infants with sustained and clinically apparent eosinophilia rather than transient mild eosinophil elevations. Finally, the relatively small number of NEC cases and the absence of formal oral food challenge or systematic re-challenge limit interpretation of NEC-specific associations and prevent definitive confirmation of suspected cow’s milk protein–related gastrointestinal disease. Accordingly, these findings should be interpreted as exploratory and hypothesis-generating rather than confirmatory.

In conclusion, gestational- and postnatal-age–adjusted eosinophilia was associated with feeding intolerance and more severe gastrointestinal manifestations in preterm infants identified using a fixed eosinophil cutoff. The concentration of all NEC cases within the eosinophilic group suggests that peripheral eosinophilia may reflect an increased susceptibility to severe intestinal inflammation in this population. However, eosinophilia appears unlikely to be a primary driver of feeding intolerance and instead may represent an accompanying immune signal emerging during the clinical course of gastrointestinal disease. When interpreted alongside gestational maturity, symptom timing, and feeding history, eosinophil counts may provide contextual supportive information in selected infants with persistent feeding intolerance or NEC-like presentations, particularly when alternative inflammatory gastrointestinal processes are being considered. Nevertheless, given the retrospective single-center design and exploratory nature of this study, prospective validation is required before eosinophilia can be incorporated into clinical assessment frameworks for feeding intolerance in neonatal intensive care practice.

## Supplementary Information

Supplementary Material 1.

## Data Availability

The datasets generated and/or analyzed during the current study are not publicly available due to patient confidentiality and institutional data protection policies but are available from the corresponding author upon reasonable request and with permission of the institutional ethics committee.

## Abbreviations

Abbreviation: Full Term
CI: Confidence Interval
CMPA: Cow’s Milk Protein Allergy
ESPGHAN: European Society for Paediatric Gastroenterology, Hepatology and Nutrition
FPIES: Food Protein–Induced Enterocolitis Syndrome
FI: Feeding Intolerance
GA: Gestational Age
IgE: Immunoglobulin E
IQR: Interquartile Range
NEC: Necrotizing Enterocolitis
NICU: Neonatal Intensive Care Unit
OR: Odds Ratio
PROM: Premature Rupture of Membranes
SD: Standard Deviation
SPSS: Statistical Package for the Social Sciences
