# Supplementary material for: Age-adjusted eosinophilia in preterm infants with feeding intolerance: a retrospective cohort study
Source: BMC Gastroenterol. 2026 Jul 22;26:662. doi: 10.1186/s12876-026-05093-w (PMC13673835; doi:10.1186/s12876-026-05093-w)
Supplement: Supplementary file 1 [file 12876_2026_5093_MOESM1_ESM.docx]

**Supplementary Table S1. G**estational- and postnatal-age–adjusted upper reference limits for eosinophil counts adapted from Henry and Christensen [14]

| **Category** | **Upper reference limit for eosinophils (×10⁹/L)** |
| --- | --- |
| **Gestational age at birth** |  |
| **<24 weeks** | 0.8–0.9 |
| **24–28 weeks** | 0.8–0.9 |
| **28–32 weeks** | 0.9–1.0 |
| **32–36 weeks** | 1.0–1.2 |
| **>36 weeks** | 1.2–1.4 |
|  |  |
| **Postnatal age** |  |
| **Days 1–7** | 1.1–1.3 |
| **Days 8–14** | 1.2–1.4 |
| **Days 15–21** | 1.4–1.6 |
| **Days 22–28** | 1.6–1.7 |
| **Days 29–40** | 1.2–1.5 |

Values are approximate upper reference intervals summarized and adapted from Henry and Christensen [14] for methodological clarification purposes.
